# Discovering large conserved functional components in global network alignment by graph matching

**DOI:** 10.1186/s12864-018-5027-9

**Published:** 2018-09-24

**Authors:** Yuanyuan Zhu, Yuezhi Li, Juan Liu, Lu Qin, Jeffrey Xu Yu

**Affiliations:** 1 School of Computer Science, Wuhan University, Bayi Road, Wuhan, 430072 China; 2 Centre of Quantum Computation and Intelligent Systems, University of Technology, Sydney, Australia; 3 The Chinese University of Hong Kong, Hong Kong, China

**Keywords:** Protein-protein interaction network, Graph theory, Graph matching

## Abstract

**Background:**

Aligning protein-protein interaction (PPI) networks is very important to discover the functionally conserved sub-structures between different species. In recent years, the global PPI network alignment problem has been extensively studied aiming at finding the one-to-one alignment with the maximum matching score. However, finding large conserved components remains challenging due to its NP-hardness.

**Results:**

We propose a new graph matching method GMAlign for global PPI network alignment. It first selects some pairs of important proteins as seeds, followed by a gradual expansion to obtain an initial matching, and then it refines the current result to obtain an optimal alignment result iteratively based on the vertex cover. We compare GMAlign with the state-of-the-art methods on the PPI network pairs obtained from the largest BioGRID dataset and validate its performance. The results show that our algorithm can produce larger size of alignment, and can find bigger and denser common connected subgraphs as well for the first time. Meanwhile, GMAlign can achieve high quality biological results, as measured by functional consistency and semantic similarity of the Gene Ontology terms. Moreover, we also show that GMAlign can achieve better results which are structurally and biologically meaningful in the detection of large conserved biological pathways between species.

**Conclusions:**

GMAlign is a novel global network alignment tool to discover large conserved functional components between PPI networks. It also has many potential biological applications such as conserved pathway and protein complex discovery across species. The GMAlign software and datasets are avaialbile at https://github.com/yzlwhu/GMAlign.

**Electronic supplementary material:**

The online version of this article (10.1186/s12864-018-5027-9) contains supplementary material, which is available to authorized users.

## Background

In many areas of bioinformatics, the generated data can be modeled as graphs such as gene co-expression networks, protein-protein interaction (PPI) network, etc. Thus, graph theory is becoming an important tool for biological network analysis. Integration of genetic-interaction data and protein-protein interaction (PPI) networks can reveal functional dependencies involved in cellular processes, including flagellum assembly, envelope integrity, and protein quality control [1]. For PPI networks whose nodes represent proteins and edges represent interactions between proteins, network alignments for comparative analysis were particularly explored [2]. Several methods have been proposed to find a mapping between nodes of two given PPI networks, to maximize the number of aligned proteins and conserved interactions to find more similar substructures. The alignment between PPI networks can help to discover the evolutionarily conserved pathways or protein complexes [3] and detect functional orthologs across species [4]. Thus, it can be applied to predicting function of unannotated proteins [5], understanding the mechanisms of human diseases [6], reproducing a rooted phylogenetic tree [7], and other areas.

Early literatures show that the sequence alignment problem has been extensively explored. However, as proved in [8], comparing DNA and protein sequences can only uncover limited information. As biotechnological advances yield more PPI data [9, 10] with complementary functional slices of the cell [11, 12], network alignment becomes more attractive with potentials in discovering more information from the topology. The inherent part of network alignment is to solve the subgraph isomorphism problem, which is NP-complete. Therefore, heuristics were studied to obtain approximate alignment results. Some local network alignment methods were developed to reveal conserved components like pathways or protein complexes between species, such as PathBLAST [13], Graemlin [14], and MaWISh [15]. PathBLAST [13] were proposed to find pathway alignments between two PPI networks with high scores and none false-positives interactions in the path. Graemlin [14] measures a module by the ratio of evolutionary constraint probability to no constraint probability, and takes phylogenetic relationships between the species into account. MaWISh [15] extends some concepts in sequence alignment such as match, mismatch and gap, and models it as a maximum weight induced subgraph problem where the structure similarity is measured based on the evolutionary events. However, the one-to-many mapping may lead to the ambiguous of the alignment [5]. Further studies explored multiple alignment based on pairwise alignment [8, 16]. Multiple alignment can indicate duplications of genes, but they are often biologically implausible [17]. Hence, most of the studies focus on the pairwise global alignment to maximize the overall matching between networks. In the following, we mainly introduce pairwise global network aligners.

One of the pioneers for global network alignment is IsoRank [17], which is based on the idea that two proteins from different networks should be matched when their neighbors are well matched. Analogous to the PageRank algorithm from Google, such intuition is modeled as an eigen-decomposition problem. PATH [18] and GA [19] model network alignment as a convex-concave programming problem, and they gradually match similar proteins to discover more conserved interactions. PATH increases the weight of the concave relaxation gradually by following the path of the solutions created. GA first finds an initial solution and then chooses a matching in the direction of a gradient objective function iteratively. The family of GRAAL algorithms [2, 5, 7, 20] are a collection of network aligners based on graphlet statistics. Newer methods in the GRAAL family usually adopt better heuristic strategies. L-GRAAL [2] uses integer programming and Lagrangian relaxation to optimize the number of proteins and the interaction functional conservations at the same time. NATALIE [21] generalizes the quadratic assignment problem based on integer linear programming, and then uses improved Lagrangian relaxation to obtain strong upper and lower bounds. GHOST [22] measures the topological similarity of proteins based on the graph spectrum and finds the alignment based on local search. NETAL [23] adds the pair of matched nodes with the largest score greedily based on both sequence information and topological structures, and the topological score can be updated dynamically. SPINAL [24] first constructs initial similarity matrix for all pairs based on local neighbourhood, and then iteratively grows a locally improved solution subset to produce the final one-to-one matching. HubAlign [25] first aligns proteins that are topologically important and then gradually match the whole network. MAGNA [26] and its extension MAGNA++ [27] align PPI network based on the genetic algorithm.

Although the global network alignment problem has been extensively explored, it remains challenging in achieving a both topologically and biologically meaningful result due to the lack of clarity of the relationship between topology and sequence. Several approaches [2, 18, 19, 23] have tried combining them together, but cannot find both topologically and biologically high quality stable matching, which leads to limited application in revealing functionally conserved components.

### Our contribution

In this paper, we propose a new global network aligner GMAlign, which can successfully combine both topology information and sequence information in a better way to produce the alignment with larger size and find more functional conserved components. GMAlign is an approach with two-stages, inspired by the graph matching method for graphs without labels [28]. In the first stage, GMAlign selects some pairs of important proteins as anchors by combining topological information and sequence information, and then obtains an initial matching by expanding from the anchors. In the second stage, GMAlign refines the initial matching to obtain suboptimal matchings iteratively based on the vertex cover. We compare GMAlign with the state-of-the-art methods, and find that it can significantly outperform existing methods in many aspects as follows.

1. GMAlign can produce larger alignment measured by edge-correctness (EC), and find bigger and denser common connected subgraphs measured by the Largest Common Connected subgraph in terms of the nodes (LCC for the evaluation of size) and edges (LCCe for the evaluation of density).
2. GMAlign achieves high biological quality in the alignment compared to other up-to-date aligners, as measured by functional consistency (FC) and average functional similarity (AFS).
3. GMAlign can find large conserved components that are both structurally and functionally meaningful, i.e., detecting large conserved sub-structures in biological pathways across species.
4. GMAlign stably outperforms existing algorithms on PPI network pairs. It can not only work well on graph pairs with general size but also can work well on the largest and densest network pair (i.e., yeast and human) where some of other aligners cannot even obtain the results in reasonable time.

## Methods

### Problem definition

We use a simple undirected graph *G*=(*V*,*E*) to model a PPI network, where a node in *u*∈*V* represents a protein and an edge (*u*,*v*)∈*E* represents the interaction between proteins *u* and *v*. Usually, the sequence information is attached to each protein, which can be considered as the node label, and a PPI network thus can be considered as a graph with node labels. Now we give the problem definition of the global network alignment.

Given two PPI networks, *G*_1_=(*V*_1_,*E*_1_) and *G*_2_=(*V*_2_,*E*_2_) with |*V*_1_|≤|*V*_2_|, a global alignment *f*:*V*_1_→*V*_2_ is a one-to-one mapping from the nodes in *G*_1_ to the nodes in *G*_2_. The global network alignment aims to find a mapping to maximize the sequence similarities of aligned proteins and the number of conserved interactions. We model the global PPI network alignment problem as graph matching, which aims to find a matching *M* between *G*_1_ and *G*_2_ according to the mapping relationship *f*, i.e., *M*={(*u*,*f*(*u*))∣*u*∈*V*_1_,*f*(*u*)∈*V*_2_}. The quality of a matching *M* can be measured by the following score function:

1 $$ score(M)=\sum_{(u_{1},v_{1}), (u_{2},v_{2})\in M}\frac{s_{(u_{1},v_{1}),(u_{2}, v_{2})}}{2},  $$

2 $$ s_{\left(u_{1},v_{1}\right),\left(u_{2},v_{2}\right)}=e_{u_{1},u_{2}}\times e_{v_{1},v_{2}} \times seq\left(u_{1},v_{1}\right) \times seq\left(u_{2},v_{2}\right)  $$

where *e*_*u*,*v*_=1 if *u* and *v* are connected, and *e*_*u*,*v*_=0 otherwise. *seq*(*u*,*v*) can be any of the sequence similarity scores between *u* and *v*. $s_{\left (u_{1},v_{1}\right),\left (u_{2},v_{2}\right)}$ is the similarity of two matched edges, including both sequence similarity and topological similarity. Thus, the problem of global network alignment is formulated as finding a matching to maximize the *score*(*M*). If we do not consider the sequence similarity, this problem can be reduced to finding maximum common subgraph, which is also NP-hard.

### GMAlign method

There are two stages in the GMAlign algorithm, matching construction and matching refinement. In the first stage, we first identify anchors followed by an expansion to other nodes to find an initial matching. In the second stage, we gradually refine the initial matching to a locally optimal matching based on the vertex cover.

#### Similarity scores

We propose a novel similarity measure to evaluate the node similarity *S*(*u*,*v*) by integrating multiple similarities, including topological similarity *S*_*t*_, degree similarity *S*_*d*_, and sequence similarity *S*_*seq*_.

##### Topological similarity.

The topological similarity *S*_*t*_ of two nodes *u* and *v* is evaluated in the context of their topological structures. To compute the topological similarity, we consider not only the local topological similarity *S*_*l*_, which describes how similar they are regarding the topological structures around them, but also the global topological similarity *S*_*g*_, which describes how similar they are regarding the whole topological structures of two graphs.

(1) *Local topological similarity*. First, for a node *v* in *G*=(*V*,*E*), we define its *k*-neighbourhood (*k*≥0) as *N*_*k*_(*v*)={*u*|*u* can reach *v* in *k*-hops}. The *k*-neighbourhood subgraph of *v* in *G* is defined as the induced subgraph over *N*_*k*_(*v*)∪{*v*} in *G*, which is denoted as $g_{v}^{k}$. The node set is denoted as $V\left (g_{u}^{k}\right) $ and the edge set is denoted $E\left (g_{u}^{k}\right) $. We can measure the local topological similarity of *u*∈*V*_1_ and *v*∈*V*_2_ by comparing their *k*-neighborhood subgraphs. Specifically, suppose that *d*(*u*) is the degree of node *u*∈*V*_1_, and *d*(*v*) is the degree of node *v*∈*V*_2_. Let *d*_1,1_,*d*_1,2_,⋯ and *d*_2,1_,*d*_2,2_,⋯ are the degree sequences of *N*_*k*_(*u*) and *N*_*k*_(*v*) respectively sorted in the non-increasing order. Let *n*_min_= min{|*N*_*k*_(*u*)|,|*N*_*k*_(*v*)|}. Then we can compute the local topological similarity of *u*∈*V*_1_ and *v*∈*V*_2_ as

3 $$ S_{l}\left (u,v\right) \,=\, \frac {\left(n_{\min}+1+D\left(u,v\right)\right)^{2}} {\left(\left | V\left(g_{u}^{k}\right)\right |\,+\,\left |E\left(g_{u}^{k}\right)\right |\right) \times \left(\left | V\left(g_{v}^{k}\right)\right |\,+\,\left | E\left(g_{v}^{k}\right)\right |\right)},  $$

4 $$  D\left(u,v\right) = \frac {\min\left \{d(u),d(v)\right\}+\sum_{i=1}^{n_{\min}} \min\left \{d_{1,i},d_{2,i}\right \}} {2}.  $$

where in Eq. (4), min{*d*(*u*),*d*(*v*)} is the ideal number of common neighbor edges when we match nodes *u* and *v*, and $\sum _{i=1}^{n_{\min }} \min \left \{d_{1,i},d_{2,i}\right \}$ is the ideal number of common edges when we match nodes in *N*_*k*_(*u*) with nodes in *N*_*k*_(*v*).

Based on above equation, we can derive that *S*_*l*_(*u*,*v*) has good properties that can effectively capture the local topology as follows (see proofs in Additional file 1).

1) 0≤*S*_*l*_(*u*,*v*)≤1. Especially, *S*_*l*_(*u*,*v*)=1 if $g_{u}^{k}$ is graph isomorphic to $g_{v}^{k}$, and *u* is matched to *v* in the optimal matching of $g_{u}^{k}$ and $g_{v}^{k}$.

2) $S_{l}\left (u,v\right)=\frac {\left | V\left (g_{u}^{k}\right)\right |+\left | E\left (g_{u}^{k}\right)\right |} {\left |V\left (g_{v}^{k}\right)\right |+\left |E\left (g_{v}^{k}\right)\right |}$, if $g_{u}^{k}$ is subgraph isomorphic to $g_{v}^{k}$, and *u* matches *v* in the optimal matching of $g_{u}^{k}$ and $g_{v}^{k}$.

3) $S_{l}\left (u,v \right) \geq \frac {\left (\left | V \left (mcs\left (g_{u}^{k},g_{v}^{k}\right)\right)\right |+\left | E\left (mcs\left (g_{u}^{k},g_{v}^{k}\right)\right)\right |\right)^{2}} {\left (\left | V\left (g_{u}^{k}\right)\right |+\left | E \left (g_{u}^{k}\right)\right |\right)\times \left (\left | V\left (g_{v}^{k}\right)\right |+\left | E \left (g_{v}^{k}\right)\right |\right)}$, where $mcs\left (g_{u}^{k},g_{v}^{k}\right)$ is the maximum common subgraph of $g_{u}^{k}$ and $g_{v}^{k}$, which is an optimal matching.

(2) *Global topological similarity*. The global topological similarity is inspired by the graph spectral theory, which can represent and distinguish structural properties of graphs by the eigenvalues and eigenvectors of its adjacency matrices. The intuition is that two isomorphic graphs will have the same eigenvalues and eigenvectors of their adjacency matrices. The earliest representative study is [29] proposed by Umeyama, which is recently improved by Knossow et al. [30]. Let *A* be the adjacency matrix for a graph *G* with *n* nodes, where *A*(*u*_1_,*u*_2_)=1 if (*u*_1_,*u*_2_)∈*E*, and *A*(*u*_1_,*u*_2_)=0 otherwise. Let *D* be the diagonal degree matrix where $D\left (u_{1},u_{1}\right)=\sum _{\left (u_{1},u_{2}\right) \in E} A\left (u_{1},u_{2}\right)$. The Laplacian matrix of *G* is defined as *L*=*D*−*A*. Suppose *L*_1_ and *L*_2_ are the Laplacian matrices of *G*_1_ and *G*_2_ with *n* nodes respectively. Let the eigenvalues of *L*_1_ and *L*_2_ be *α*_1_≥*α*_2_≥⋯≥*α*_*n*_ and *β*_1_≥*β*_2_≥⋯≥*β*_*n*_ respectively. As *L*_1_ and *L*_2_ are symmetric and positive-semidefinite, we can decompose them as $L_{1}=U_{1} \Lambda _{1} U_{1}^{T}$ and $L_{2}= U_{2} \Lambda _{2} U_{2}^{T}$, where *U*_1_ and *U*_2_ are orthogonal matrices, and *Λ*_1_=*diag*(*α*_*i*_) and *Λ*_2_=*diag*(*β*_*i*_). If *G*_1_ and *G*_2_ are isomorphic, there exists a permutation matrix *P* such that $PU_{1} \Lambda _{1} U_{1}^{T} P^{T}=U_{2} \Lambda _{2} U_{2}^{T}$. Let $P=U_{2} {D}' U_{1}^{T}$ where *D*^′^=*diag*(*d*_1_,⋯,*d*_*n*_) and *d*_*i*_∈{+1;−1} accounts for the sign ambiguity in the eigendecomposition. When *G*_1_ and *G*_2_ are isomorphic, the optimal permutation matrix is *P* which maximizes $tr\left (P^{T} \bar {U_{2}}\bar {U_{1}}^{T} \right)$, where $\bar {U_{1}}$ and $\bar {U_{2}}$ are matrices whose elements are the absolute values of elements in *U*_1_ and *U*_2_ respectively. When the numbers of nodes in *G*_1_ and *G*_2_ are not the same, we only choose the largest *c* eigenvalues where *c*= min{|*V*(*G*_1_)|,|*V*(*G*_2_)|}. Let ${\bar {U_{1}}}'$ and ${\bar {U_{2}}}'$ be the first *c* columns of $\bar {U_{1}}$ and $\bar {U_{2}}$ respectively, the global similarity matrix can be obtained as

5 $$ S_{g}={\bar{U_{1}}}'{\bar{U_{2}}}'^{T}.  $$

Here, *S*_*g*_(*u*,*v*)∈[0,1] is the global topological similarity between *u*∈*V*_1_ and *v*∈*V*_2_.

Based on the local topological similarity *S*_*l*_ and the global topological similarity *S*_*g*_, we measure the topological similarity *S*_*t*_ for nodes *u* and *v* by combining them together as follows.

6 $$ S_{t} \left (u, v \right) = S_{l} \left (u,v \right) \times S_{g} \left (u,v\right), \forall u \in V_{1}, v \in V_{2}.  $$

##### Degree similarity.

In addition to the topological structure *S*_*t*_ around nodes *u* and *v*, we also consider their similarity based on degrees of themselves, which is defined as

7 $$ S_{d} \left(u,v\right) = \frac {\min\left \{d(u),d(v)\right \}} {\max\left \{d(u),d(v)\right \}}.  $$

Both topological similarity *S*_*t*_ and degree similarity *S*_*d*_ are measures to capture graph structure. Thus, we use the structure similarity *S*_*str*_ to integrate them by adding a balancing parameter *θ*∈[0,1].

8 $$ S_{str}(u,v) = \left (1 - \theta \right)\times S_{t}(u,v) + \theta \times S_{d}(u,v).  $$

##### Sequence similarity.

In addition, we also consider their sequence similarity when we match two nodes *u* and *v*, which is defined as

9 $$ S_{seq}\left (u, v \right)= \frac{seq\left (u,v\right)}{\max_{i \in V_{1}, j \in V_{2}} seq\left (i,j \right)},  $$

where *seq*(*u*,*v*) can be any of the sequence similarity scores (in this article, we use both log of BLAST’s e-values and BLAST’s bit-scores). *S*_*seq*_(*u*,*v*) is in the range of [0,1] after the normalization.

Finally, we obtain the overall similarity score of *u*∈*V*_1_ and *v*∈*V*_2_ by integrating the structure similarity *S*_*str*_ and the sequence similarity *S*_*seq*_ together by a balancing parameter *α*∈[0,1].

10 $$ S(u,v) = \left (1 - \alpha \right) \times S_{str}(u,v)+\alpha \times S_{seq}(u,v).  $$

#### Matching construction

To construct an initial matching, we first choose some important node pairs with high similarity scores and large degrees as anchors, and then we expand from the anchors to match the rest of the nodes gradually.

We select anchors according to the following two conditions:

1) min{*d*(*u*),*d*(*v*)}≥*δ*$\left (\delta = \max \left \{ \frac {2\times |E_{1}|} {\left |V_{1}\right |}, \frac {2\times \left | E_{2}\right |} {\left |V_{2}\right |}\right \}\right)$.

2) *S*(*u*,*v*)≥*τ*, where *τ* is a threshold and generally *τ*≥0.5. The detailed method of automatically tuning a suitable *τ* is given in Additional file 1.

The anchors selected based on above criteria play two important roles. First, they contribute a large number of edges to the matching *M* because they are similar with each other. Second, they can be important references in the matching process due to their high degrees.

Algorithm 1 shows the process of matching construction. First, we compute score *S*(*u*,*v*) for all *u*∈*V*_1_,*v*∈*V*_2_ in line 2, followed by the sort of pairs in the decreasing order of *S*(*u*,*v*) and the selection of matched anchors pairs in lines 3–5. Then we expand the matching by adding anchors in *A* to *M*. For every matched pair (*u*,*v*)∈*M*, we put all the *N*(*u*)×*N*(*v*) pairs into a candidate queue *Q*, where *Q* is sorted in the decreasing order of their expansion similarity in lines 6–7. It is a variant of *S*(*u*,*v*) obtained by excluding the global similarity as the expansion is in a local manner. Then we iteratively remove the pair (*u*,*v*) with largest similarity from *Q* in line 9. If both *u* and *v* have not been matched before, we add (*u*,*v*) to *M* and put *N*(*u*)×*N*(*v*) into *Q* for further consideration in lines 10–11. The loop ends when *Q* is empty.

#### Matching refinement

The heuristics used to obtain the initial matching *M* cannot guarantee the optimality of *M*. We further study how to refine the initial matching to get better matching results. The main idea is that each time we check some part of the matching to see if we can directly obtain an improved matching by avoiding the exhaustive search over the matching space.

For a graph *G*, we define its vertex cover as a subset of nodes *C*⊆*V*, such that *u*∈*C* or *v*∈*C* for each edge (*u*,*v*)∈*E*. The complement of a vertex cover *I*=*V*−*C* is an independent set of the graph. In other words, *C* is the set of nodes that covers all the edges in the graph, while in *I*=*V*−*C* there exists no edge. This also means that a node in *C*⊆*V*_1_ can possibly have many edges to cover or possibly have many matched edges with graph *G*_2_.

We use *R*_1_ and *R*_2_ to represent the matched nodes in graphs *G*_1_ and *G*_2_ respectively in the matching *M*. For any (*u*,*v*)∈*M*, we have *u*∈*R*_1_ and *v*∈*R*_2_. Given a vertex cover *C*⊆*V*(*G*_1_), we use *H*_1_ to denote *C*∩*R*_1_, and use *H*_2_=*M*[*H*_1_] to denote the corresponding matched part of *H*_1_ in *R*_2_. The nodes of *G*_1_ are divided into three parts *H*_1_, *C*−*H*_1_ and *V*_1_−*C*. According to the definition of vertex cover, the nodes in *H*_1_ may lead to good matches, which are thus be excluded in the refinement. *C*−*H*_1_ should be included in the refinement as the nodes in *C*−*H*_1_ have not been matched to any nodes. The independent set *V*_1_−*C* is also included in the refinement because the contribution of matched edges for each node in this set will not affect each other. Then by excluding *H*_1_, we can compute a refined matching *M*^∗^(*H*_1_) for *G*_1_ and *G*_2_ based on the initial matching *M* and vertex cover *C*⊆*V*(*G*_1_) as follows.

First of all, we build a bipartite graph *G*_*b*_, where node set *V*_1_−*H*_1_ is on one side and nodes set *V*_2_−*H*_2_ is on the other side. For any *u*∈*V*_1_−*H*_1_ and *v*∈*V*_2_−*H*_2_, we add an edge (*u*,*v*) to *G*_*b*_ and its weight can be computed as

11 $$ w\left(u,v\right)=\left | M\left[N(u)\cap H_{1}\right]\cap \left(N(v)\cap H_{2} \right)\right |.  $$

where *w*(*u*,*v*) can be consider as the number of matched edges if we match *u* in *G*_1_ with *v* in *G*_2_.

Then, based on the Hungarian algorithm, we can find the maximum weighted bipartite matching *M*_*b*_ of *G*_*b*_ such that the total weight of edges in *M*_*b*_ is maximized. Thus, a new *M*^∗^(*H*_1_) can be derived as

12 $$ M^{*} \left(H_{1}\right) = \left(M\cap\left(H_{1}\times H_{2}\right)\right)\cup M_{b},  $$

where *H*_1_×*H*_2_ is the cartesian product of *H*_1_ and *H*_2_ which includes all the pairs (*u*,*v*) for all *u*∈*H*_1_ and *v*∈*H*_2_. The optimality of *M*^∗^(*H*_1_) has been proved in [28].


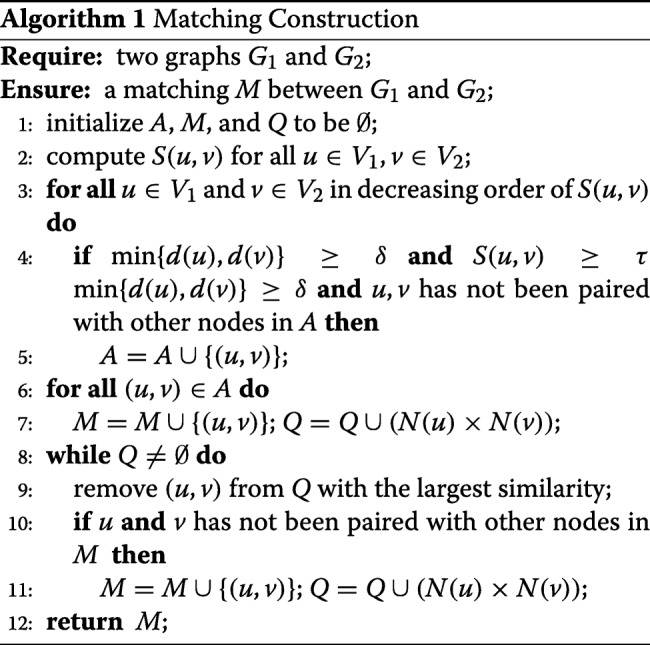


If the most of nodes in the selected vertex cover *C* are not well matched, or *M* itself is already an optimal matching in the solution space ${\mathcal {M}}$, it is possible that *M*^∗^(*H*_1_) is not better than *M*. The reason is that the mismatched nodes are excluded by the vertex cover *C*. To solve such problem, we propose two strategies. The first is to *C* smaller, such that more mismatched nodes can be included to refine. The second is to refine current matching using different vertex covers iteratively so that every mismatched node has a chance to be refined.


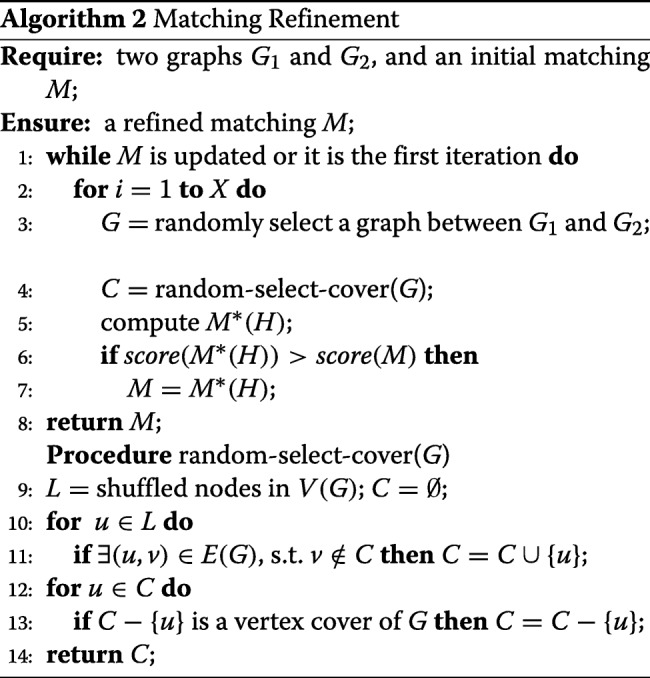


To find a smaller *C*, a straightforward method is to find a minimum vertex cover of *G*_1_. However, it is impractical because of the following two reasons. 1) Finding a minimum vertex cover of a graph is NP-hard. 2) In a minimum vertex cover, the mismatched nodes do not have a chance to be included to refine. To avoid these drawbacks, we use a minimal vertex cover, because 1) a minimal vertex cover is easy to find, and 2) the number of different minimal vertex covers for a graph is much larger than the number of different minimum vertex covers.

We give the random selection process of a minimal vertex cover of graph *G* in Algorithm 2 in lines 9–14. First, we shuffle all nodes in the graph and put them into a list *L*, such that any permutation of *V*(*G*) has the same probability in *L*. Then, we find a vertex cover of *G* by adding nodes in *L* one by one. We only add a node into the vertex cover if it can contribute at least one edge to the edges covered so far.

We implement this operation as follows. We maintain the number of uncovered edges for each node in the graph, which is initialized to be its degree. When we add a new node into the cover set, we will first skip it if the number of its uncovered edges is 0. Otherwise, we add it into the cover set, and traverse its neighbours. For each neighbour, we decrease the number of its uncovered edges by 1. The total complexity of this process in lines 10–11 is *O*(|*E*(*G*)|), because every edge in *G* is visited at most once. We remove some useless nodes to guarantee the minimality of the current vertex cover in lines 12–13, such that the removal of these nodes does not influence any edge currently covered.

Algorithm 2 shows the whole process of matching refinement. We choose the vertex cover *C* of either *G*_1_ or *G*_2_ with the same probability so that each graph has a chance to be refined in line 3. We refine the matching by different vertex covers multiple times, so that every mismatched node will have a chance to be included to refine. Such process repeated iteratively to update current matching until no improvement can be acheived in a certain number of iterations. We try *X* times to find a new random vertex cover *C*, and update the current matching if *M*^∗^(*H*) is a better matching in each iteration. Here, we set *X* to be a constant (≥1) to avoid the case that only one bad cover will terminate the whole process.

The complexity of the whole algorithm including construction and refinement is *O*(*m*×*n*^3^) where *n* and *m* are the minimum numbers of nodes and edges in these two graphs respectively. The complexity is mainly dominated by eigendecomposition and maximum weighted bipartite matching methods, and can be largely reduced if alternative node similarity computation and approximate bipartite matching methods are applied [28].

### Datasets

The dataset we used in this paper is the same as that in L-GRAAL [2]. It contains eight PPI networks of different organisms from BioGRID database with the largest number of known physical interactions [9]. They are: HS (*H*.*sapiens* with 13,276 nodes and 110,528 edges), SC (*S*.*cerevisiae* with 5831 nodes and 77,149 edges), AT (*A*.*thaliana* with 5897 nodes and 13,381 edges), DM (*D*.*melanogaster* with 7937 nodes and 34,753 edges), CE (*C*.*elegans* with 3134 nodes and 5428 edges), MM (*M*.*musculus* with 4370 nodes and 9116 edges), SP (*S*.*pombe* with 1911 nodes and 4711 edges), and RN (*R*.*norvegicus* with 1657 nodes and 2330 edges). The details of these datasets are listed in Table 1. The physical interactions in BioGRID can be either direct (e.g., from yeast-two-hybrid) or indirect (e.g., from affinity capture). The protein sequences and GO annotations are extracted from NCBI’s Entrez Gene database [31]. Note that we only retrieve experimentally validated GO annotations (i.e. GO term evidence codes: IPI, IGI, IMP, IDA, IEP, TAS and IC), from which we further removed the annotations inferred from the PPIs (code IPI). We will validate our alignment results by detecting conserved pathways between species. We download the pathways in the species from the KEGG database [32]. As stated in [33], many aligners have memory issues when dealing with the pair of the two largest networks yeast (SC) and human (HS). Thus, we will first give comparison results based on the $\binom {6}{2} = 15$ pairs of networks DM, AT, MM, CE, SP and RN, which can be solved by all the aligners, and then we give the results of SC and HS for aligners that can run to completion later.

**Table 1:** The datasets of PPI networks

Networks	Nodes	Edges	Average degree
RN	1657	2330	2.812
CE	3134	5428	3.464
MM	4370	9116	4.1728
AT	5897	13,381	4.538
SP	1911	4711	4.930
DM	7937	34,753	8.7579
HS	13,276	110,528	16.651
SC	5831	77,149	26.462

### Evaluation measures

#### Topological measures

*f* is the mapping from the *G*_1_=(*V*_1_,*E*_1_) to *G*_2_=(*V*_2_,*E*_2_) with |*V*_1_|≤|*V*_2_|. Let *f*(*V*_1_)={*f*(*v*)∈*V*_2_|*v*∈*V*_1_} and *f*(*E*_1_)={(*f*(*u*),*f*(*v*))∈*E*_2_|(*u*,*v*)∈*E*_1_}. We evaluate the topological quality of the an alignment by the measures in the following.

##### Edge correctness (EC).

EC is the ratio of the number of conserved edges under the mapping *f* to the number of edges in the small network, which can be computed as follows [5].

$$EC=\frac {\left |f\left(E_{1}\right)\right |} {\left |E_{1}\right |}. $$

##### Largest common connected subgraph (LCC and LCCe).

The largest common connected subgraph in an alignment consists of nodes and edges denoted by *V*_*m*_ and *E*_*m*_, respectively. LCC is calculated as the fraction of nodes in the largest connected subgraph in an alignment, which is computed by

$$LCC=\frac {|V_{m}|} { |V_{1}|}. $$

In addition to the size, the density of LCC is also another important property for graphs. As stated by [7], bigger and denser subgraphs can give more insight into common structure of the networks. Meanwhile, bigger and denser subgraphs may be more biologically important [34]. For example, Bader and Spirin [35, 36] have shown that a dense PPI subgraph may correspond to a vital protein complex. Hence, we propose a new measure to evaluate the density of LCC by the fraction of edges in the largest connected subgraph in an alignment, which is computed by

$$LCCe = \frac {|E_{m}|} { |E_{1}|}. $$

##### Symmetric sub-structure score (S^3^).

S^3^ is the fraction of conserved edges between the smaller network and the sub-network from the larger network induced by the alignment to measure how the mapped regions are topologically similar. It is defined as

$$S^{3}=\frac {\left |f\left(E_{1}\right)\right |} {\left | E_{1}\right |+\left | E\left(G_{2}\left(f\left(V_{1}\right)\right)\right)\right |-\left | f\left(E_{1}\right)\right |}. $$

#### Biological measures

##### Functional consistency (FC).

We use gene ontology (GO) terms to measure the functional consistency of two aligned proteins [25]. GO terms describe the biological properties of a protein such as the Molecular Function (MF), Cellular Component (CC), and Biological Process (BP). Proteins with similar GO terms usually are functionally similar. We use the fraction of aligned proteins with common GO terms with respect to the size of the smaller network to evaluate the biological significance of an alignment. The larger the fraction is, the more biologically meaningful the alignment is. Suppose there are *G*_≥*a*_ aligned proteins having at least *a* common GO terms. Then we can calculate *FC*_≥*a*_ as follows:

$$FC_{\geq a}=\frac {G_{\geq a}} {|V_{1}|}, a \in N_{+}. $$

##### Average of functional similarity (AFS).

AFS is the semantic similarity of the GO terms, which mainly depends on the distance between them in the ontology. We can calculate the functional similarity in each category of BP, MF and CC. The semantic similarity is computed by the Resnik semantic similarity [37] with the best-match average mixing strategy. We use *S*_*c*_(*u*,*v*) to represent the GO functional similarity of proteins *u* and *v* in category *c* (i.e., BP, MF or CC). Then, we measure the average of functional similarity of the entire alignment in category *c*, *AFS*_*c*_, by the sum of the semantic similarities of all mapped proteins, divided by the number of annotated proteins in the smaller network. That is:

$$AFS_{c} = \frac {\sum_{u \in V_{1}}^{} S_{c}\left(u,f(u)\right)} {\left | V_{1}\right |}, c \in \left \{ BP, MF, CC \right \}. $$

#### Detecting conserved pathways

In addition to the above separated structural and biological measures, we further evaluate the quality of alignments by a higher-level similarity measure that can combine both the functional and structural information, the conserved pathways between networks. In fact, many biological pathways with similar functions exist in different organisms [3]. The experimentally validated biological pathways are provided in the KEGG PATHWAY database [32]. A pathway is a set of proteins, whose name consists of two parts (e.g., hsa03010), the name of a species (hsa for Homo sapiens) and an pathway ID (03010). Pathways with the same ID in different species have similar biological functions. We show the biological meaning of the alignment results by retrieving the experimentally proven protein interactions in pathways from the APID dataserver [38].

**Fig. 1:**
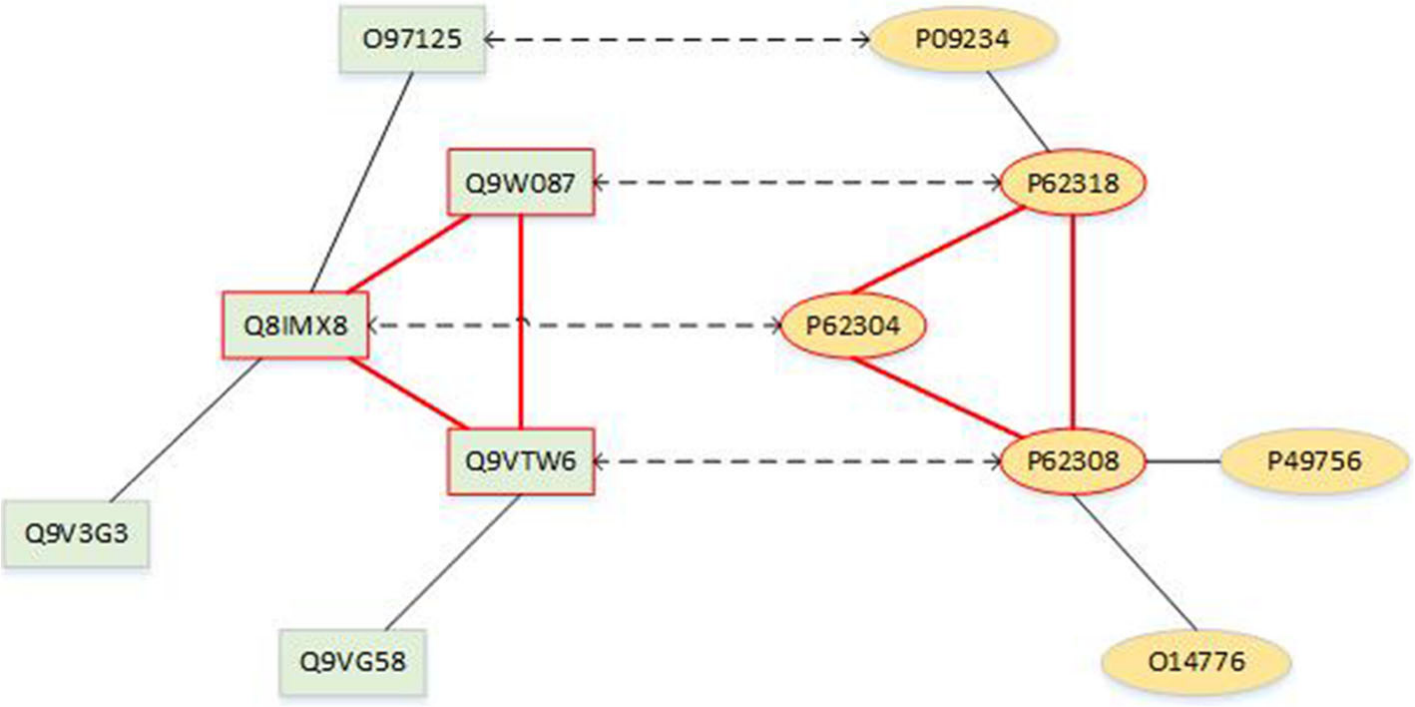
The example of hsa03040 and dme03040 aligned by GMAlign. The left network is part of dme03040 while the right is part of hsa03040. The dotted line denotes mapping relationship produced by GMAlign. The sub-structure marked red is the final common sub-structure between hsa03040 and dme03040 found by GMAlign and experimentally proved by APID dataserver

Here, we give the procedure of detecting conserved pathways between species. First, we find mapped KEGG pathways through alignment results and thus we get the common sub-structure in the mapped pathways. Then, we retrieve the proven common sub-structure between the mapped pathways by the APID dataserver [38], where all protein interactions are proven experimentally in existing publications. Let us take hsa03040 and dme03040 for example. The hsa03040 is a pathway taken from the human (HS) KEGG database while dme03040 is taken from the fruit fly (DM) KEGG database. They have the same number 03040 which means they share similar biological function. In Fig. 1, the left network is part of the induced network of proteins in dme03040 while the right is part of hsa03040. The dotted line represents the mapping relationship produced by GMAlign. Then we retrieve the real interactions between them which were experimentally proven by the APID dataserver [38]. The sub-structure marked red is the final real common sub-structure of the dme03040 and hsa03040 pathways. That is, in this example GMAlign finds 3 common nodes and 3 common interactions between dme03040 pathway of the fruit fly and hsa03040 pathway of human.

## Results

We compare our algorithm GMAlign with GHOST [22], NETAL [23], SPINAL [24], HubAlign [25], MI-GRAAL [7], L-GRAAL [2], and MAGNA [27], which are state-of-the-art methods that are publicly available. For MI-GRAAL, we investigated the performance of the combinations of its five similarity measures, and we repeated the alignment process 15 times for each combination because of its randomness to find alignments of the best biological and topological quality. Following the recommendation in paper [24], we use mode II in SPINAL. We use the improved version MAGNA++ [27] instead of MAGNA to optimize the S^3^ score, on a population size of 2000 over 15,000 generations as L-GRAAL does. For all the evaluated aligners, we set other parameters at their default values. For aligners such as GMAlign and L-GRAAL that can produce alignments using topology or sequence information by balancing parameter *α*∈[0,1], we sample the balancing parameters from 0 to 1 with step size of 0.1. We will also evaluate robustness of the different methods by adjusting the parameter in the same way. We set *θ*=0.5 in GMAlign to balance the topological structure and self-degree, neighborhood size *k* to 2, and iteration number *X* to 5 in GMAlign to achieve good performance stably on all the network pairs. All the algorithms run on a PC with an Intel Core I7-4790 CPU at 3.6GHz with 64GB memory.

### Topological analysis

#### General size PPI network alignment

**Fig. 2:**
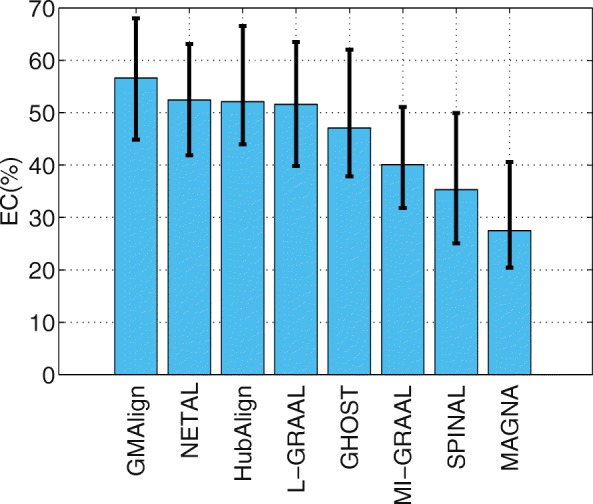
The alignment results for PPI network with general size. All the comparison are based on the $\binom {6}{2}=15$ pairs of networks among GMAlign, HubAlign, L-GRAAL, NETAL, GHOST, MI-GRAAL, SPINAL and MAGNA. The results for Edge Correctness

**Fig. 3:**
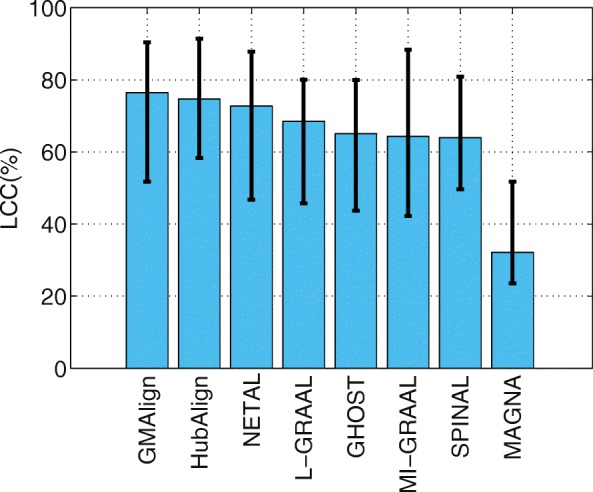
The alignment results for PPI network with general size. All the comparison are based on the $\binom {6}{2}=15$ pairs of networks among GMAlign, HubAlign, L-GRAAL, NETAL, GHOST, MI-GRAAL, SPINAL and MAGNA.The results for nodes of Largest Common Connected subgraph

**Fig. 4:**
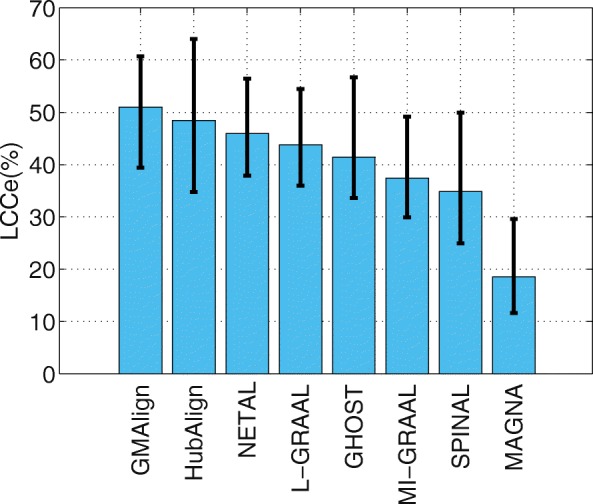
The alignment results for PPI network with general size. All the comparison are based on the $\binom {6}{2}=15$ pairs of networks among GMAlign, HubAlign, L-GRAAL, NETAL, GHOST, MI-GRAAL, SPINAL and MAGNA.The results for edges of Largest Common Connected subgraph

First, we evaluate the topological quality of the alignments on the $\binom {6}{2} = 15$ network pairs of general size. As shown in Fig. 2, GMAlign can produce the largest alignment with EC of 56.62*%*, while the EC for NETAL [23], HubAlign [25] and L-GRAAL [2] are 52.47, 52.10, and 51.61*%* respectively. We can see that GMAlign has significant advantage in finding a bigger size in the alignment. As [2] does, we also measure the statistical significance of the obtained EC scores using the standard model of sampling without replacement proposed in [5] (We give the detailed formula in Additional file 1). We can see that, the results produced by GMAlign are statistically significant, as the probability of obtaining similar or higher values by chance is always smaller than 0.05. Meanwhile, Fig. 3 shows the LCC produced by all the algorithms, we can see that GMAlign, HubAlign and NETAL produce the least fragmented network alignments, with LCC of 76.43, 74.69, and 72.71*%*, respectively. In addition, as Fig. 4 shows, GMAlign can discover the most number of conserved edges in the LCC subgraph of the alignment with LCCe of 50.97*%*, while the LCCe are only 48.43*%* for HubAlign and 45.95*%* for NETAL. This shows that GMAlign is also capable of finding bigger and denser common connected subgraph that is biologically important for PPI networks.

**Fig. 5:**
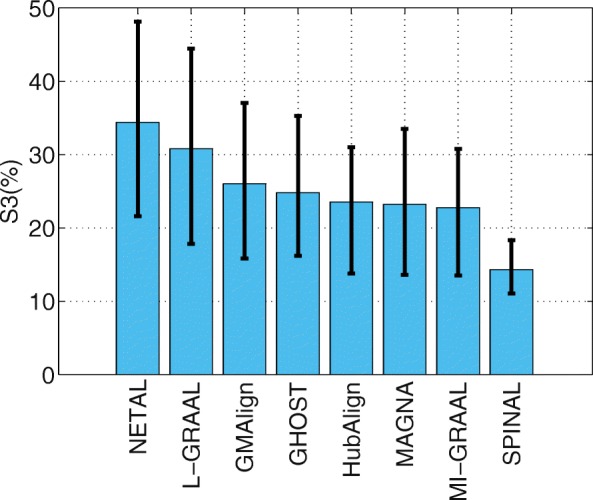
The alignment results for PPI network with general size. All the comparison are based on the $\binom {6}{2}=15$ pairs of networks among GMAlign, HubAlign, L-GRAAL, NETAL, GHOST, MI-GRAAL, SPINAL and MAGNA.The results for Symmetric Sub-structure Score

Now we evaluate the measure S^3^. As shown in Fig. 5, NETAL [23] achieves the highest value at 34.39*%*.L-GRAAL [2] and GMAlign follow behind it, with values of 30.82*%* and 26.02*%* respectively. As we know, S^3^ is a penalization when aligning sparse regions with dense regions. However, such penalization is not very reasonable when it is necessary to map a sparse network to a dense network, especially when the scales and densities of the 6 evaluated PPI networks are different. Thus, we believe that S^3^ is only a reference to show the density similarity of the mapped regions and cannot be considered a principle measure to evaluate the topological quality. The detailed results in Figure 2-5 are given in Additional file 2.

Overall, GMAlign, NETAL [23], HubAlign [25], and L-GRAAL [2] outperform all the other methods in terms of the topological quality on the general PPI networks. Among these methods, GMAlign can find the largest alignment and discover the biggest and densest common connected subgraphs, which implies that that GMAlign has a higher possibility to find a biologically meaningful sub-structure, such as pathways and complexes.

#### Yeast-human PPI network alignment

We evaluate the algorithms on two large networks, human (HS) and yeast (SC). Since not all the aligners can finish the alignment for these two large networks in reasonable time, we only report the results for the capable aligners.

First, as shown in Table 2, GMAlign still can find larger alignments of 30.17*%* for EC, bigger and denser common connected subgraph of 99.49*%* for LCC and 30.17*%* for LCCe. It enhances the conclusion in MI-GRAAL [7] that there exists a surprising amount of common PPI network topology between human and yeast. As reported in [7], for yeast network with 2390 nodes and 16,127 edges and human network with 9141 nodes and 41,456 edges, MI-GRAAL finds that 77.7*%* proteins in the yeast had a high-confidence PPI subnetwork that is fully contained within the human high-confidence PPI subnetwork. In this paper, we use the datasets which are far larger than those in MI-GRAAL, and GMAlign, HubAlign [25] and L-GRAAL [2] finds a higher percentage of nodes shared by yeast and human. Meanwhile, a high percentage of shared edges (30.17*%*) was found for the first time by GMAlign.

**Table 2:** Network alignment results for yeast and human^1^

Method	EC (*%*)	LCC (*%*)	LCCe (*%*)	S^3^(*%*)
GMAlign	**30.17**	99.49	**30.17**	**16.89**
HubAlign	27.46	**99.74**	27.46	15.20
L-GRAAL	15.74	98.03	15.67	11.91
MAGNA	10.74	74.43	10.70	8.26

^1^The best cases are show in boldface

Second, surprisingly, Table 2 shows that GMAlign can also find similar sub-structures in the two networks with S^3^ of 16.89*%*, while L-GRAAL only achieves 11.91*%*. This is the first time that GMAlign not only produces alignment with a larger EC, LCC, and LCCe, but also finds more sub-structures with similar density. We believe that the underlying reason is HS and SC have similar densities.

As we can see all above, GMAlign has excellent ability in producing larger size alignment and finding bigger and denser common connected subgraphs. Moreover, GMAlign has the potential in finding same parts with similar density in the two networks, which depends on the properties of the matched networks such as degree distributions.

#### Balancing topology and sequence information

We investigate the relationship between the topology information and sequence information on the $\binom {6} {2}=15$ pairs of networks. We compare GMAlign with HubAlign [25] and L-GRAAL [2] by varying *α* from 0 to 1, and compute the average value of all the pairwise alignments on each value of *α*.

**Fig. 6:**
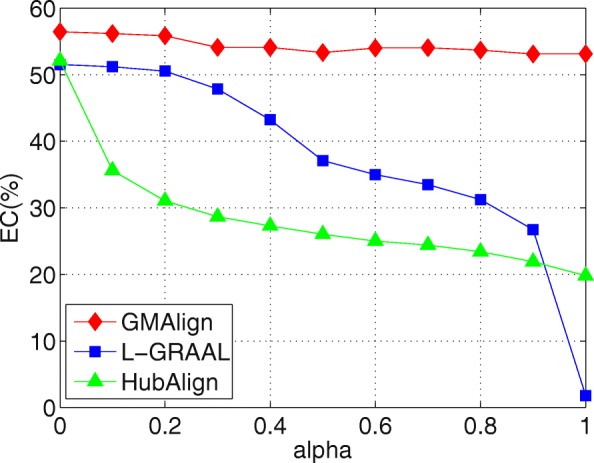
Balancing sequence and topology information. All the comparison are based on $\binom {6}{2}=15$ pair of networks among GMAlign, L-GRAAL and HubAlign when *α* are varied from 0 to 1. The results for Edge Correctness

**Fig. 7:**
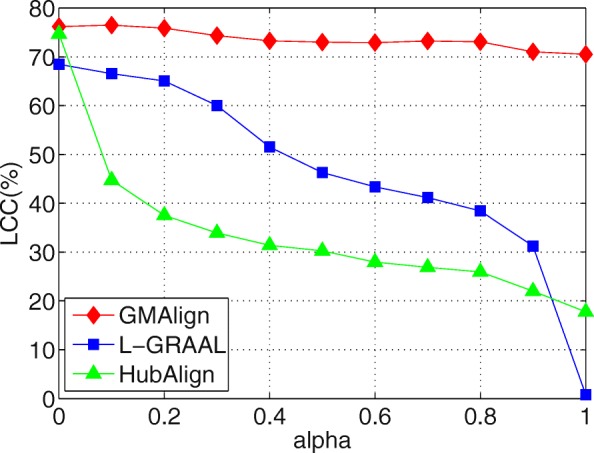
Balancing sequence and topology information. All the comparison are based on $\binom {6}{2}=15$ pair of networks among GMAlign, L-GRAAL and HubAlign when *α* are varied from 0 to 1.The results for nodes of Largest Common Connected subgraph

**Fig. 8:**
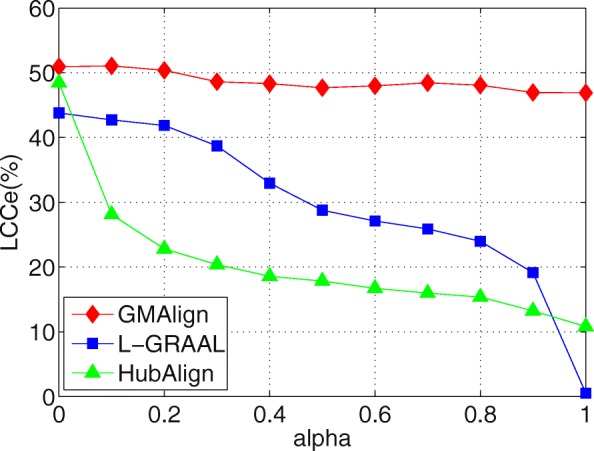
Balancing sequence and topology information. All the comparison are based on $\binom {6}{2}=15$ pair of networks among GMAlign, L-GRAAL and HubAlign when *α* are varied from 0 to 1. The results for edges of Largest Common Connected subgraph

**Fig. 9:**
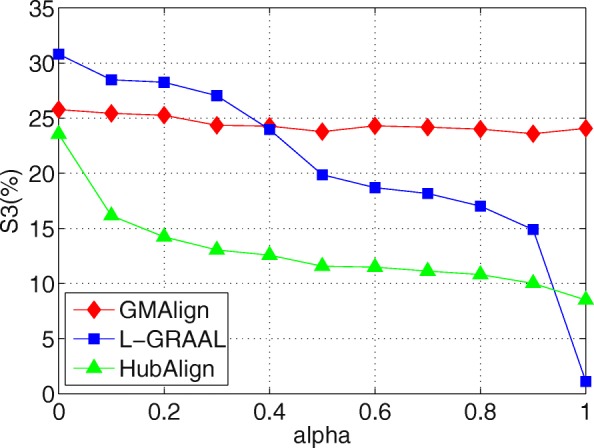
Balancing sequence and topology information. All the comparison are based on $\binom {6}{2}=15$ pair of networks among GMAlign, L-GRAAL and HubAlign when *α* are varied from 0 to 1.The results for Symmetric Sub-structure Score

First, as shown in Figs. 6, 7 and 8, when we vary *α* from 0 to 1, GMAlign is always stable while HubAlign and L-GRAAL and drastically decrease when transferring from topology information to sequence information. Figure 9 shows that for S^3^, GMAlign is outperformed slightly by L-GRAAL for small *α*, but it outperforms L-GRAAL for large *α* because of its stability. In fact, there might be hidden connections between topology information and sequence information, and the difficulty is how to combine them naturally without too much conflict. We combine them at the bottom level of node similarity more naturally while the other two methods combine them at a very high level with respect to the whole node set and edge set.

Overall, GMAlign can produce larger size alignment and find bigger and denser common connected subgraphs robustly under different parameter settings. All the algorithms achieve the best topological matching quality when we only use topology information, which is also consistent with the declaration that topology plays a more important role than sequence for uncovering functionally conserved interactions [2].

### Biological analysis

The biological analysis is based on the alignments generated. For methods with tunable parameters between topology and sequence information, we only used topology information. The reason is that very few mapping nodes are generated when involving sequence information, and topology plays a more important role than sequence as declared in [2].

#### Functional consistency analysis

We measure the Functional Consistency (FC) based on the fraction of aligned proteins sharing common GO terms. We show the FC score for alignment based on the yeast (SC) and human (HS) PPI networks in Table 3. Both GMAlign and HubAlign can align more nodes that shares GO terms. Up to 20.31*%* aligned nodes have at least one GO term shared for GMAlign, while the fraction for L-GRAAL is only 13.67*%*. GMAlign and HubAlign can even align some nodes that share more than 5 GO terms.

**Table 3:** Functional consistency of the alignment for yeast and human^1^

No. of shared GO terms	GMAlign	HubAlign	MAGNA	L-GRAAL
≥1	**20.31**	20.17	14.49	13.67
≥2	3.5	**4.1**	2.45	2.01
≥3	0.38	**0.57**	0.26	0.26
≥4	0.07	**0.14**	0.09	0.03
≥5	**0.03**	**0.03**	0.02	0

^1^The best cases are show in boldface

Similar experiments are also conducted on the $\binom {6}{2}=15$ pairs of networks (see Table 4), and we can obtain the same conclusion as above. One thing that must be noticed is that FC reflects the ability of aligners in finding functionally conserved proteins regardless of the topological structure. For PPI networks, topological structure may play a more important role in biological function, because proteins do not work alone but work together. Hence, when we compare different aligners, we can refer to FC but not rely on it although GMAlign has competitive FC values.

**Table 4:** Functional consistency of alignments for $\binom {6}{2}=15$ pair of networks^1^

No. of shared GO terms	GMAlign	HubAlign	GHOST	NETAL	MI-GRAAL	L-GRAAL	MAGNA	SPINAL
≥1	**8.8**	8.56	8.31	8.08	7.82	7.66	7.16	4.20
≥2	1.3	**1.32**	1.21	1.09	1.13	1.02	0.91	0.44
≥3	0.18	0.18	**0.21**	0.13	0.18	0.19	0.08	0.05
≥4	0.05	0.04	**0.06**	0.02	**0.06**	0.04	0.03	0.02
≥5	0.01	**0.02**	**0.02**	0.01	0.01	0.01	0.01	0

^1^The best cases are show in boldface

#### Functional similarity analysis

**Fig. 10:**
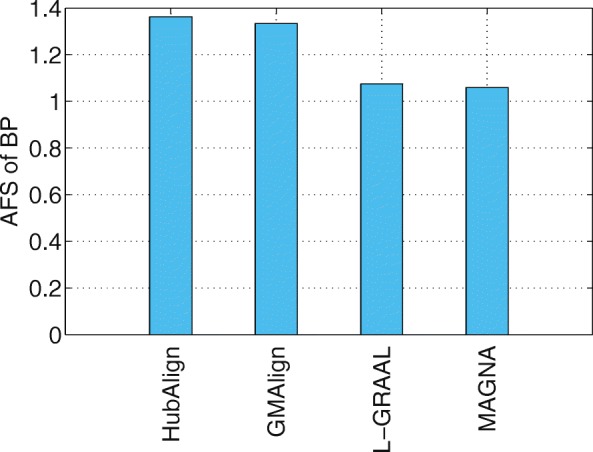
The average functional similarity (AFS) of the alignment of yeast and human. All the comparison are only for aligners that can produce results in reasonable time (GMAlign, HubAlign, L-GRAAL and MAGNA). The average functional similarity (AFS) for category BP

**Fig. 11:**
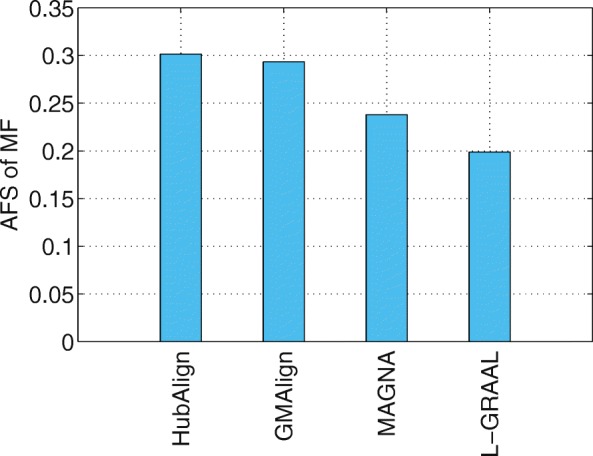
The average functional similarity (AFS) of the alignment of yeast and human. All the comparison are only for aligners that can produce results in reasonable time (GMAlign, HubAlign, L-GRAAL and MAGNA). The average functional similarity (AFS) for category MF

**Fig. 12:**
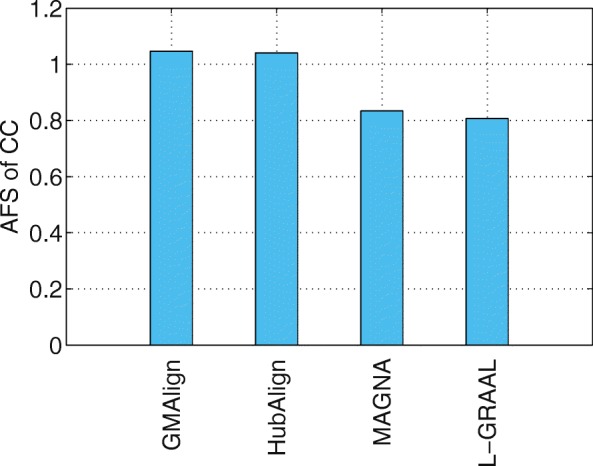
The average functional similarity (AFS) of the alignment of yeast and human. All the comparison are only for aligners that can produce results in reasonable time (GMAlign, HubAlign, L-GRAAL and MAGNA). The average functional similarity (AFS) for category CC

**Fig. 13:**
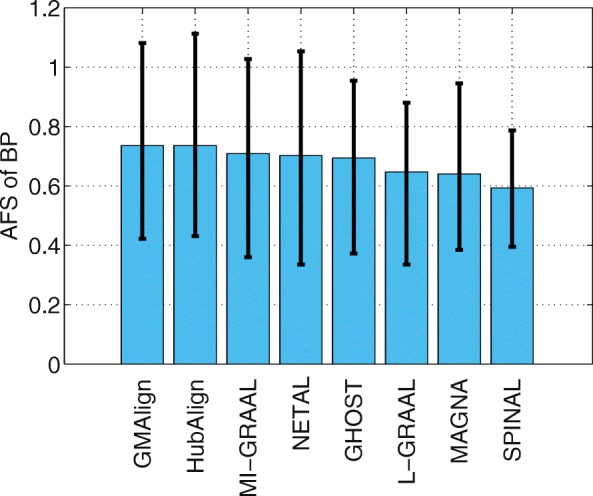
The average functional similarity (AFS). All the comparison are based on $\binom {6}{2}=15$ pairs of networks among GMAlign, HubAlign, L-GRAAL, NETAL, GHOST, MI-GRAAL, SPINAL and MAGNA. The average functional similarity (AFS) for category BP

**Fig. 14:**
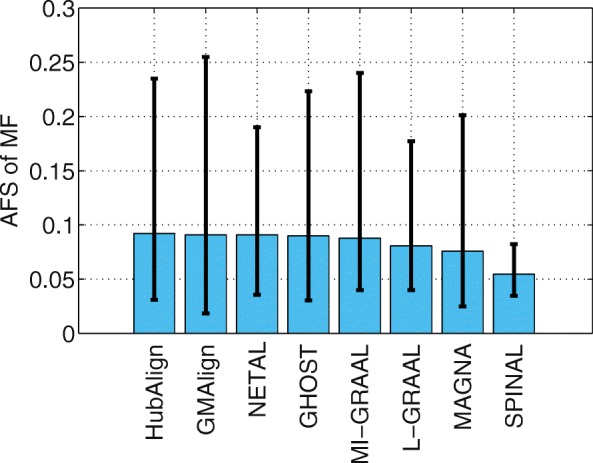
The average functional similarity (AFS). All the comparison are based on $\binom {6}{2}=15$ pairs of networks among GMAlign, HubAlign, L-GRAAL, NETAL, GHOST, MI-GRAAL, SPINAL and MAGNA. The average functional similarity (AFS) for category MF

**Fig. 15:**
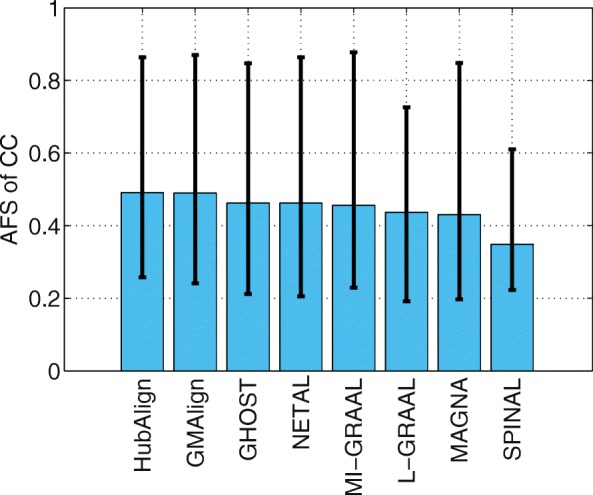
The average functional similarity (AFS). All the comparison are based on $\binom {6}{2}=15$ pairs of networks among GMAlign, HubAlign, L-GRAAL, NETAL, GHOST, MI-GRAAL, SPINAL and MAGNA. The average functional similarity (AFS) for category CC

Functional similarity (AFS) provides an alternative way to describe the biological quality of an alignment, which is calculated based on the semantic similarity of the GO terms associated with the mapped proteins. The AFS score in each category of BP, MF and CC in the ontology for the yeast-human network alignment is displayed in Figs. 10, 11 and 12. Similar results for the alignment of $\binom {6} {2}=15$ network pairs are also provided in Figs. 13, 14 and 15.

We can see that GMAlign outperforms other aligners in terms of the AFS in the CC category with *AFS*_*CC*_ of 1.047 (see Fig. 12). Meanwhile, GMAlign and HubAlignv [25] also perform best in the BP and MF categories (see Figs. 10 and 11), with *AFS*_*BP*_ of 1.333 for GMAlign and 1.362 for HubAlign and *AFS*_*MF*_ of 0.293 for GMAlign and 0.301 for HubAlign. Similar conclusion can be made from the alignment results of $\binom {6} {2}=15$ network pairs displayed in Figs. 13, 14 and 15 and we provide all the detailed data in Additional file 3. Overall, GMAlign and HubAlign outperform all other aligners in terms of the biological quality of their alignments, and moreover, GMAlign can also achieve the best topological quality of the alignments.

### Detecting conserved pathways

We further evaluate the algorithms by the detection of functional conserved pathways on the largest PPI networks, human (SC) and yeast (HS), which have been investigated a lot in the literature [2, 23, 25, 26]. We validate our findings by only considering the protein interactions that are already experimentally proven in APID dataserver [38].

**Fig. 16:**
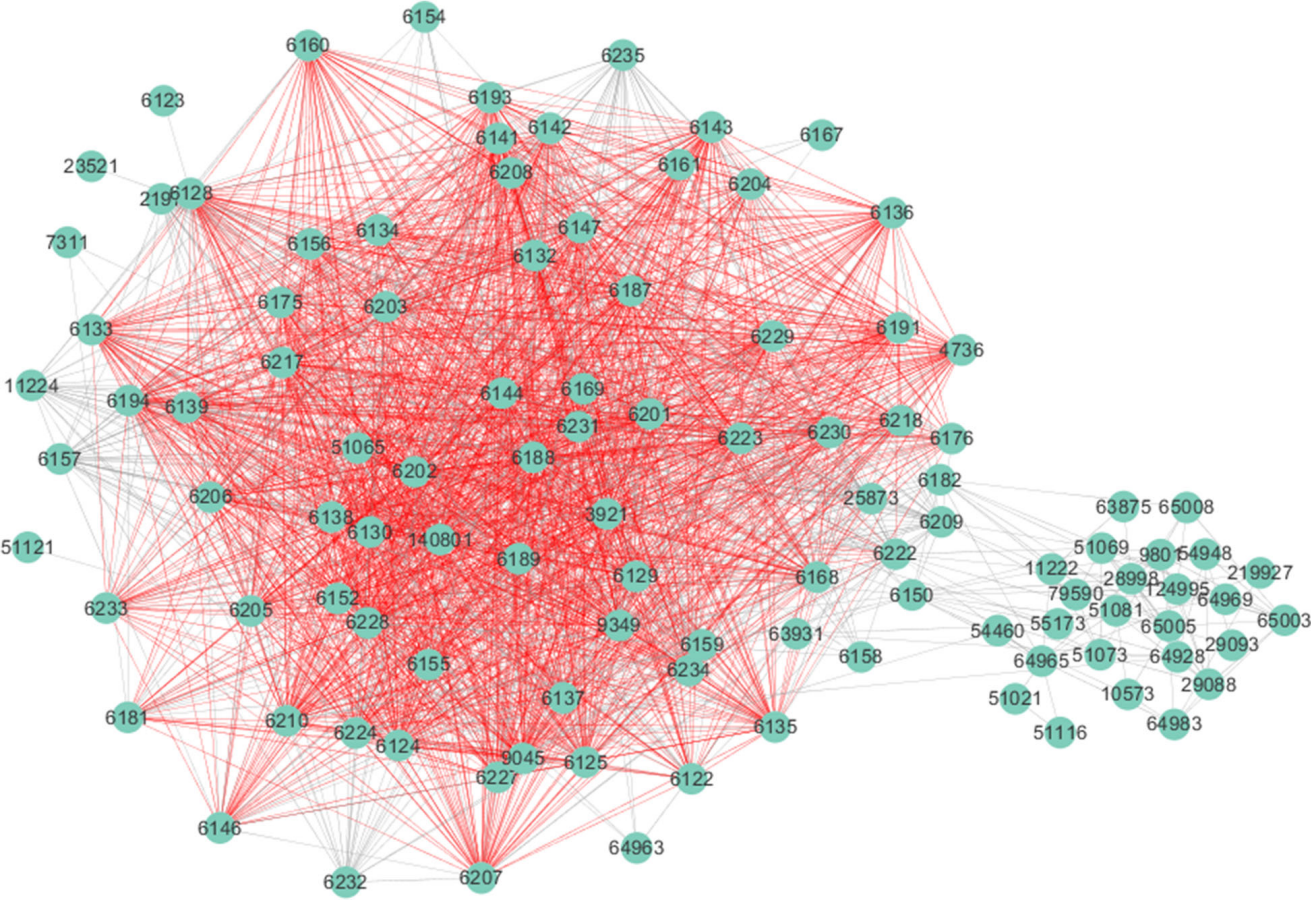
The structure of hsa03010 pathway. The common sub-structure in sce03010 pathway found by GMAlign is marked red

**Fig. 17:**
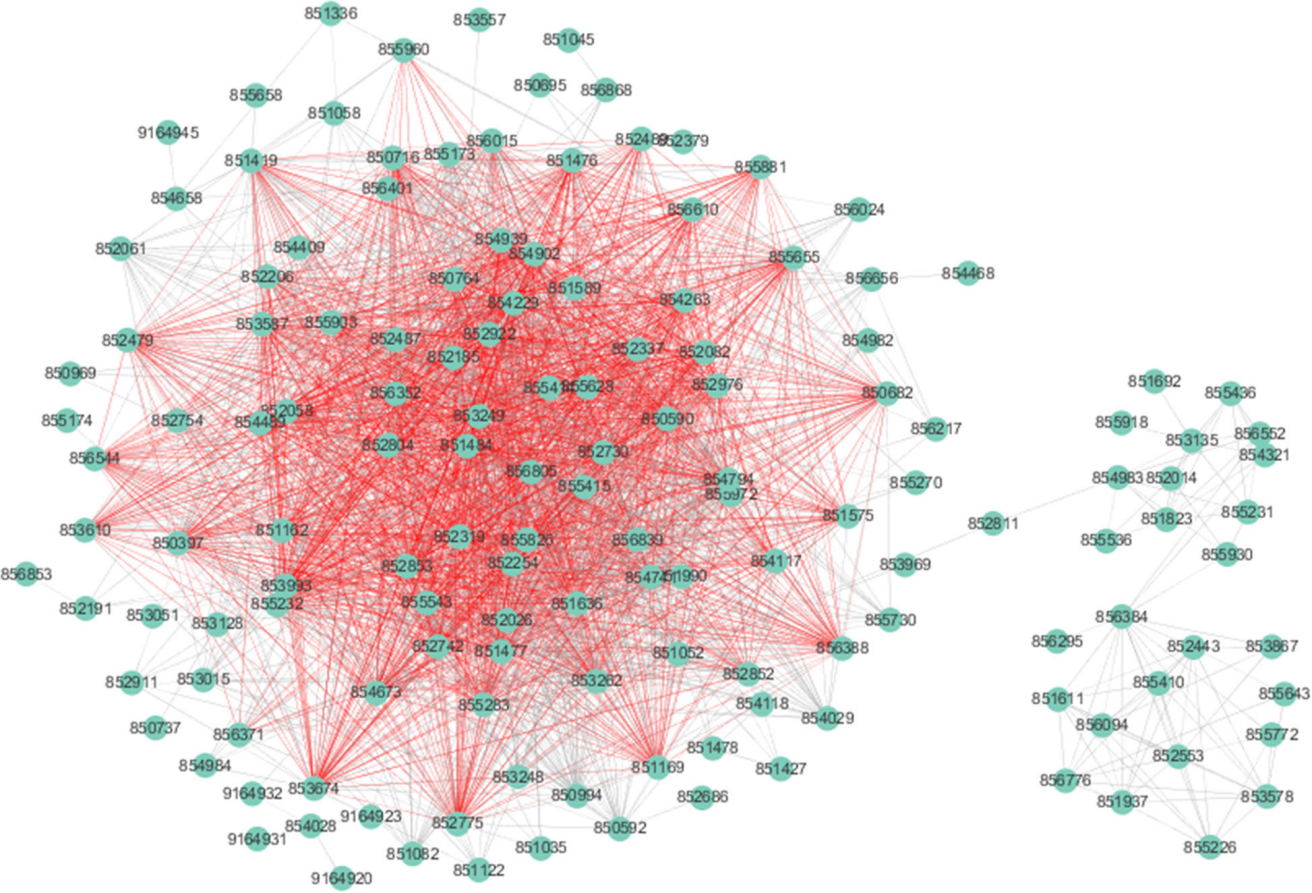
The structure of sce03010 pathway. The common sub-structure in hsa03010 pathway found by GMAlign is marked red

**Fig. 18:**
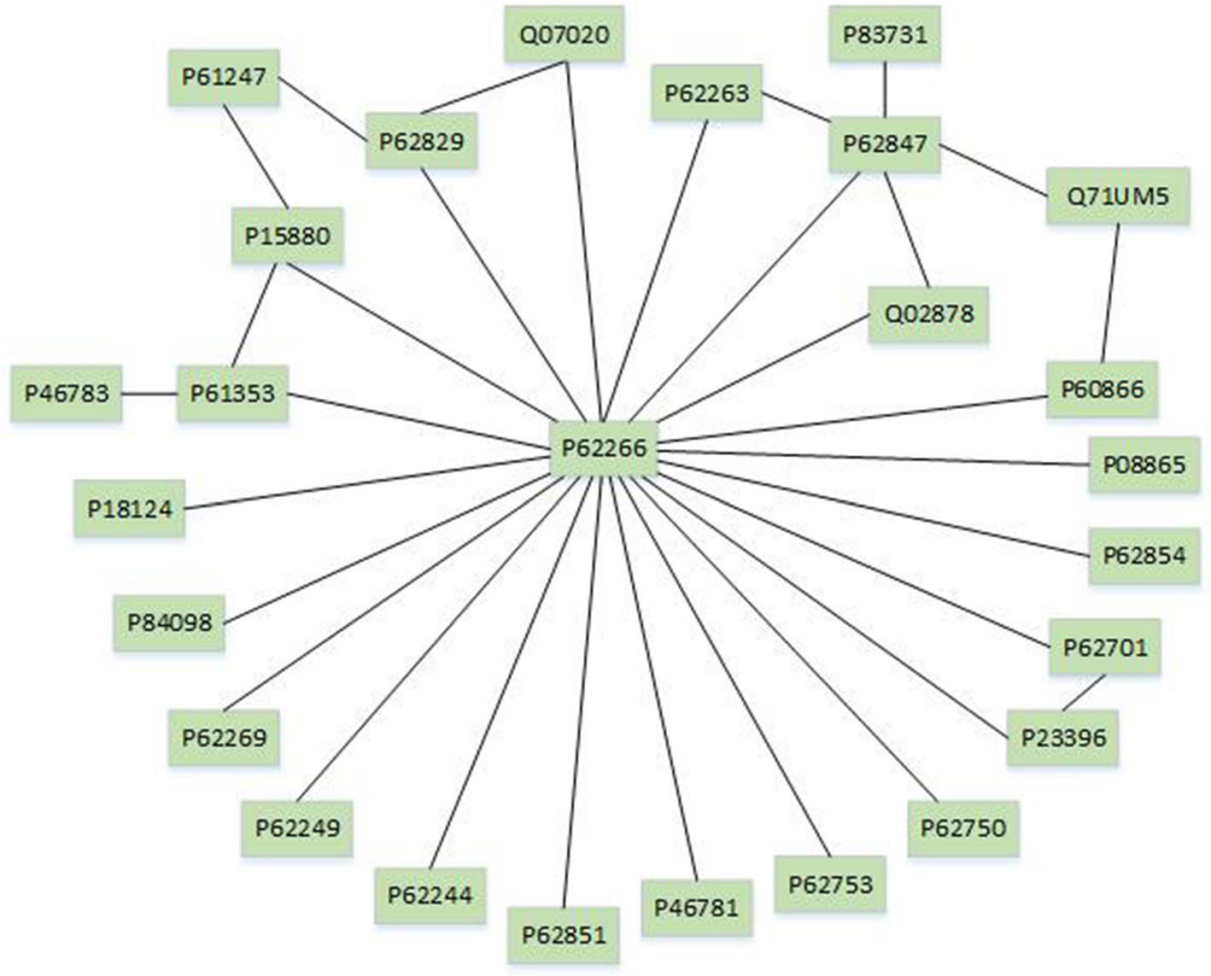
The final conserved sub-structure of hsa03010 pathway

**Fig. 19:**
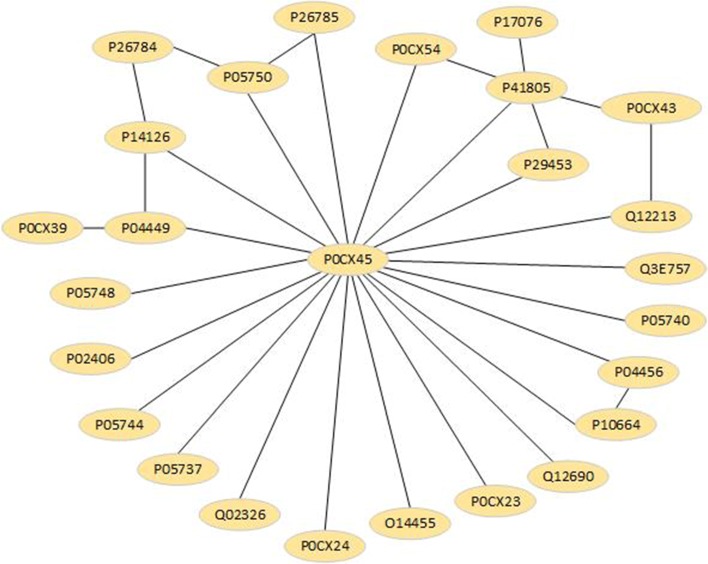
The final conserved sub-structure of sce03010 pathway

The conserved part of the sce03010 and hsa03010 pathways in the alignment obtained by GMAlign is shown in Figs. 16 and 17. Although there has been a lot of studies [39] on the relationship between the ribosome biogenesis pathway (03010) of yeast and human, it is the first time we give their mapping details in a global alignment. Figures 16 and 17 show the structure of hsa03010 pathway and the sce03010 pathway respectively with the mapped sub-structure marked red in GMAlign, where hsa03010 has 132 proteins and 1924 interactions, and sce03010 has 175 proteins and 2311 interactions. GMAlign can discover a large functional conserved sub-structure with 63 proteins and 1406 interactions (details are listed in Additional file 4), while the best competitor HubAlign [25] can only find 58 mapped proteins and 914 mapped interactions (details are listed in Additional file 5). MAGNA [27] only discovers 23 common proteins and 123 common interactions (details are listed in Additional file 6), and L-GRAAL [2] cannot even detect any common protein or interaction between hsa03010 and sce03010 (results are listed in Additional file 7). Furthermore, we validate sa03010 and sce03010 in the APID dataserver [38], and found that hsa03010 has 126 proteins and 1748 interactions experimentally proved by existing publications while sce03010 only has 165 proved proteins and 192 proved interactions. Figures 18 and 19 show the validated sub-structure of the pathways, and GMAlign finds that hsa03010 and sce03010 share a relatively complete sub-structure consisting of 26 proteins and 32 interactions proven by publications. Besides sce03010 and hsa03010, GMAlign can also discover other small conserved pathways, such as mmu05200 and hsa05200 with 4 common proteins and 3 common interactions, and dme03040 and hsa03040 with 3 common proteins and 3 common interactions, after the validation of APID dataserver, while other algorithms fails. We provide the details of conserved pathways discovered in Additional files 4, 5, 6, 7 and 8, and we can find that HubAlign and MAGNA cannot find any other conserved pathways except for the pair of yeast and human, and the conserved pathways discovered by L-GRAAL and NETAL are also smaller than those discovered by GMAlign. This result can benefit future biological studies on pathways and implies the potential of our algorithm in exploring the relationship of functional components across different species.

## Discussion

The purpose of network aligners is to find functional and structural similarities between PPI networks of different species [40]. Most existing network alignment algorithms solve this problem as an optimization problem over the convex combination of sequence and structural similarities between two networks [2, 5, 19, 25, 41]. They can generally be classified into two types according to their optimization targets: sequence similarity tendency and structural similarity tendency [33]. The sequence similarity tendency aligners usually rely too much on the similarity between two proteins, such as BLAST scores to find large conserved sub-networks. The structural similarity tendency aligners can achieve better results in discovering large conserved subgraphs but their biological accuracy needs to be improved.

It seems that most current aligners cannot combine the optimization of both sequence similarity and structural similarity very well [33]. For example, IsoRank [41] uses only a function of the node degrees as the structural similarity combined with the BLAST scores, which leads to its poor performance in finding structurally and biologically similar sub-structure. Other state-of-the-art aligners make considerable progress in considering their relationship. L-GRAAL [2] adopts graphlets to calculate the structural similarity. HubAlign [25] adopts minimum-degree heuristics based on the observation that topologically important proteins in a PPI network usually are much more conserved. MAGNA++ [27] can optimize any alignment accuracy measure but is only restricted to topological similarity measures. Other aligners, such as NETAL [23], GHOST [22] and SPINAL [24] have a similar problem.

GMAlign combines multiple similarities including both topological similarity and sequence similarity from the early alignment procedure to the refinement stage to get more meaningful topological and biological results. Our experimental results confirm that GMAlign can find bigger and denser common connected sub-structures (see Figs. 2, 3, 4 and 5), which means that there is a large probability of finding biologically meaningful structures. Moreover, we prove that GMAlign can achieve better biological quality (Figs. 10, 11 and 12 and see Figs. 13, 14 and 15). Even more, GMAlign discovers the close relationship between the sce03010 pathway and the hsa03010 pathway and gives their inner relationship which is proven using the APID dataserver [38]. Further experiments about adjusting the ratio between the topological similarity and sequence similarity (see Figs. 6, 7, 8 and 9) confirm that existing aligners are not as robust as GMAlign.

PPI network alignment is an effective method to discover the functionally conserved sub-structure between networks, which is significant for biological studies. As we discussed above, GMAlign outperformed these aligners in many aspects, but it has its own limitations in terms of efficiency. Thus, we will try to optimize the computation process and develop a parallel version of the algorithm to obtain better efficiency in future work. Moreover, we will try more biological applications to make full use of GMAlign, such as predicting protein interactions [5], detecting functional orthologs across species [4] and understanding the mechanisms of human diseases [6].

## Conclusions

In this article, we propose a new network aligner, GMAlign, which first constructs an initial matching by selecting anchor pairs, followed by a gradual expansion, and then iteratively refines current matching to a suboptimal matching based on vertex cover. We found a way to successfully combine the topology and sequence information at the level of nodes without too much conflict. Experimental comparison of GMAlign with many state-of-the-art aligners on the PPI networks from BioGRID shows that GMAlign can produce larger size alignments, and find bigger and denser common connected subgraphs. Additionally, to the best of our knowledge, this is the first time that LCCe has been proposed to evaluate the density of the largest common connected subgraph found in an alignment.

Second, GMAlign also performs well in matching functionally conserved proteins using topology information, as measured by the functional consistency and semantic similarity. This shows that GMAlign can map many protein pairs with common GO terms and higher semantic similarity.

Finally, GMAlign detects a large conserved part of the pathways across yeast and human, which shows that GMAlign can integrate sequence and topology information in a better way to find structurally and functionally meaningful components. These results will significantly benefit the biological studies on the relationship between the pathways of different species. In the future work, we will optimize the efficiency of GMAlign and explore potential applications of GMAlign on predicting protein interactions, detecting functional orthologs across species and understanding the mechanisms of human diseases.

## Additional files

Additional file 1 The supplementary materials for GMAlign. (PDF 152 kb)

Additional file 2 The detailed results of Figure 2–5 for all aligners. (XLS 7 kb)

Additional file 3 The detailed results of Figure 13–15 for all aligners. (XLS 8 kb)

Additional file 4 The results produced by GMAlign. Each pathway pair has four sheets to describe alignment results. The former two sheets represent retrieving results of two species by APID dataserver [38] respectively. The third sheet is direct alignment results of GMAlign. The forth sheet is the final conserved interactions of the pathway pair. (XLS 79 kb)

Additional file 5 The results produced by HubAlign. Each pathway pair has four sheets to describe alignment results. The former two sheets represent retrieving results of two species by APID dataserver [38] respectively. The third sheet is direct alignment results of HubAlign. The forth sheet is the final conserved interactions of the pathway pair. (XLS 59 kb)

Additional file 6 The results produced by MAGNA. Each pathway pair has four sheets to describe alignment results. The former two sheets represent retrieving results of two species by APID dataserver [38] respectively. The third sheet is direct alignment results of MAGNA. The forth sheet is the final conserved interactions of the pathway pair. (XLS 14 kb)

Additional file 7 The results produced by L-GRAAL. Each pathway pair has four sheets to describe alignment results. The former two sheets represent retrieving results of two species by APID dataserver [38] respectively. The third sheet is direct alignment results of L-GRAAL. The forth sheet is the final conserved interactions of the pathway pair. (XLS 6 kb)

Additional file 8 The results produced by MAGNA. Each pathway pair has four sheets to describe alignment results. The former two sheets represent retrieving results of two species by APID dataserver [38] respectively. The third sheet is direct alignment results of NETAL. The forth sheet is the final conserved interactions of the pathway pair. (XLS 9 kb)

## Abbreviations

APID: Agile protein interactomes dataserver
BLAST: Basic local-alignment search tool
KEGG: Kyoto encyclopedia of genes and genomes
PPI: Protein-protein interaction
