# Supplementary material for: Discovering large conserved functional components in global network alignment by graph matching
Source: BMC Genomics. 2018 Sep 24;19(Suppl 7):670. doi: 10.1186/s12864-018-5027-9 (PMC6157291; doi:10.1186/s12864-018-5027-9)
Supplement: Supplementary file 1 — The supplementary materials for GMAlign. (PDF 152 kb) [file 12864_2018_5027_MOESM1_ESM.pdf]

RESEARCH

Supplementary Material

Discovering large conserved functional components in global network alignment by graph matching

Yuanyuan Zhu[1*], Yuezhi Li[1], Juan Liu[1*] , Lu Qin[2] and Jeffrey Xu Yu[3]

1 Statistical significance of EC

When aligning two networks $G_1 = (V_1, E_1)$ and $G_2 = (V_2, E_2)$, under the stadard model of sampling without replacement, the probability p of obtaining at least k common edges by chance is the tail of the hypergeometric distribution:

$$p(k) = \sum_{i=k}^{m_2} \frac{\binom{m_2}{i}\binom{M-m_2}{m_1-i}}{\binom{M}{m_1}} \quad (1)$$

where $m_1 = |E_1|$, $m_2 = |E_2|$ and $M = |V_2| \times (|V_2| - 1)/2$ is the number of node pairs in G_2 [1].

2 Proof of properties of local topological similarity

1) $0 \leq S_l(u,v) \leq 1$. Especially, $S_l(u,v) = 1$ if g_u^k and g_v^k are isomorphic, and u is matched to v in the optimal matching of g_u^k and g_v^k.

2) $S_l(u,v) = \frac{|V(g_u^k)|+|E(g_u^k)|}{|V(g_v^k)|+|E(g_v^k)|}$, if g_u^k is subgraph isomorphic to g_v^k, and u matches v in the optimal matching of g_u^k and g_v^k.

3) $S_l(u,v) \geq \frac{\left(\left|V\left(mcs\left(g_u^k,g_v^k\right)\right)\right|+\left|E\left(mcs\left(g_u^k,g_v^k\right)\right)\right|\right)^2}{(|V(g_u^k)|+|E(g_u^k)|)\times(|V(g_v^k)|+|E(g_v^k)|)}$, where $mcs\left(g_u^k, g_v^k\right)$ is the maximum common subgraph of g_u^k and g_v^k which is an optimal matching.

For 1), it's obvious that $S_l(u,v) > 0$ holds, because both $(n_{min} + 1 + D(u,v))^2 > 0$ and $(|V(g_u^k)| + |E(g_u^k)|)(|V(g_v^k)| + |E(g_v^k)|) > 0$. $S_l(u,v) \leq 1$ can be showed as follows. Since $\min\{d(u), d(v)\} \leq d(u)$ and $\min\{d_{1,i}, d_{2,i}\} \leq d_{1,i}$, $D(u,v) \leq \frac{d(u) + \sum_{i=1}^{n_{min}} d_{1,i}}{2} = |E(g_u^k)|$. Similarly, $D(u,v) \leq |E(g_v^k)|$. By combining such two inequations with the fact that $n_{min} + 1 \leq |V(g_u^k)|$ and $n_{min} + 1 \leq |V(g_v^k)|$, we have $S_l(u,v) \leq 1$. When the two subgraphs are isomorphism, we have $n_{min} + 1 = |V(g_u^k)| = |V(g_v^k)|$ and $D(u,v) = |E(g_u^k)| = |E(g_v^k)|$. For 2), it is because when g_u^k is subgraph isomorphic to g_v^k, we have $(n_{min} + 1 = |V(g_u^k)|$ and $D(u,v) = \frac{d(u) + \sum_{i=1}^{n_{min}} d_{1,i}}{2} = |E(g_u^k)|$, which leads to $S_l(u,v) = \frac{|V(g_u^k)| + |E(g_u^k)|}{|V(g_v^k)| + |E(g_v^k)|}$. For 3), since the node number of either g_u^k or g_v^k appearing in the maximum common subgraph can never exceed the minimum node number of g_u^k and g_v^k, $|V(g_u^k, g_v^k)| \leq n_{min} + 1$. Also, $D(u,v)$ is known to be an upper bound of $|E(g_u^k, g_v^k)|$, which is proved in [2]. Thus, this inequation holds. Here, $S_l(u,v)$ is an upper bound of such similarity, if we treat the right side of the equation as an accurate similarity of two nodes based on their maximum common subgraph.

*Correspondence: yyzhu@whu.edu.cn; liujuan@whu.edu.cn
[1]Computer School, Wuhan University, Bayi Road, 430072, Wuhan, China
Full list of author information is available at the end of the article

Author details
[1]Computer School, Wuhan University, Bayi Road, 430072, Wuhan, China. [2]Centre of Quantum Computation and Intelligent Systems, University of Technology, Sydney, Australia. [3]The Chinese University of Hong Kong, Hong Kong, China.

References

1. Przulj, N., Corneil, D., Juristica, I.: Modeling interactome: Scale-free or geometric? Bioinformatics **20**, 3508–3515 (2004)
2. Raymond, J.W., Gardiner, E.J., Willett, P.I.: Rascal: Calculation of graph similarity using maximum common edge subgraphs. The Computer Journal **45(6)**, 631 (2002)
